# Isolation of a Potently Neutralizing and Protective Human Monoclonal Antibody Targeting Yellow Fever Virus

**DOI:** 10.1128/mbio.00512-22

**Published:** 2022-04-14

**Authors:** Michael P. Doyle, Joseph R. Genualdi, Adam L. Bailey, Nurgun Kose, Christopher Gainza, Jessica Rodriguez, Kristen M. Reeder, Christopher A. Nelson, Prashant N. Jethva, Rachel E. Sutton, Robin G. Bombardi, Michael L. Gross, Justin G. Julander, Daved H. Fremont, Michael S. Diamond, James E. Crowe

**Affiliations:** a Department of Pathology, Microbiology and Immunology, Vanderbilt University Medical Center, Nashville, Tennessee, USA; b Department of Pathology and Immunology, Washington University School of Medicine, St. Louis, Missouri, USA; c The Vanderbilt Vaccine Center, Vanderbilt University Medical Center, Nashville, Tennessee, USA; d Department of Chemistry, Washington University in St. Louis, St. Louis, Missouri, USA; e Institute for Antiviral Research, Department of Animal, Dairy, and Veterinary Sciences, Utah State University, Logan, Utah, USA; f Department of Biochemistry and Molecular Biophysics, Washington University School of Medicine, St. Louis, Missouri, USA; g Department of Molecular Microbiology, Washington University School of Medicine, St. Louis, Missouri, USA; h Department of Medicine, Washington University School of Medicine, St. Louis, Missouri, USA; i Department of Pediatrics, Vanderbilt University Medical Center, Nashville, Tennessee, USA

**Keywords:** monoclonal antibodies, mouse model, neutralization, vaccine, yellow fever virus

## Abstract

Yellow fever virus (YFV) causes sporadic outbreaks of infection in South America and sub-Saharan Africa. While live-attenuated yellow fever virus vaccines based on three substrains of 17D are considered some of the most effective vaccines in use, problems with production and distribution have created large populations of unvaccinated, vulnerable individuals in areas of endemicity. To date, specific antiviral therapeutics have not been licensed for human use against YFV or any other related flavivirus. Recent advances in monoclonal antibody (mAb) technology have allowed the identification of numerous candidate therapeutics targeting highly pathogenic viruses, including many flaviviruses. Here, we sought to identify a highly neutralizing antibody targeting the YFV envelope (E) protein as a therapeutic candidate. We used human B cell hybridoma technology to isolate mAbs from circulating memory B cells from human YFV vaccine recipients. These antibodies bound to recombinant YFV E protein and recognized at least five major antigenic sites on E. Two mAbs (designated YFV-136 and YFV-121) recognized a shared antigenic site and neutralized the YFV-17D vaccine strain *in vitro*. YFV-136 also potently inhibited infection by multiple wild-type YFV strains, in part, at a postattachment step in the virus replication cycle. YFV-136 showed therapeutic protection in two animal models of YFV challenge, including hamsters and immunocompromised mice engrafted with human hepatocytes. These studies define features of the antigenic landscape of the YFV E protein recognized by the human B cell response and identify a therapeutic antibody candidate that inhibits infection and disease caused by highly virulent strains of YFV.

## INTRODUCTION

Yellow fever virus (YFV), the prototype and namesake member of the family *Flaviviridae*, is a historically important human pathogen. Yellow fever (YF) disease has been described in the New World since the 1600s, and YFV was first identified in 1927 (1). The virus has caused numerous epidemics of human disease throughout the world. According to the World Health Organization, 47 countries in Africa and Central and South America currently have regions where yellow fever is endemic, and the burden of yellow fever disease during 2019 was as high as 109,000 severe cases and 51,000 deaths (2). Approximately 30% of infected individuals develop severe disease that includes hemorrhagic complications and multiorgan failure, half of whom succumb to the infection (3). Nonhuman primates serve as the primary reservoir for YFV, with mosquitoes in the *Haemagogus*, *Sabethes*, and *Aedes* genera serving as the vectors responsible for reservoir maintenance and spillover into humans, typically when humans encroach on primates’ natural ecosystems, in what is referred to as the “sylvatic cycle.” Once within the human population, YFV is spread by a different vector, the anthropophilic Aedes aegypti mosquito, in an “urban cycle” (4). Beginning in 2018, YFV epidemics began approaching coastal urban centers like Sao Paolo and Rio de Janeiro, Brazil, sparking concerns that more severe YFV epidemics may occur in the future (5).

YFV is an enveloped virus with a positive-sense, single-stranded RNA genome. The YFV genome is translated as a single polyprotein, which is posttranslationally cleaved by a combination of host and viral proteins into 3 structural (pr/M, E, and C) and 7 nonstructural (NS1, NS2A, NS2B, NS3, NS4A, NS4B, and NS5) proteins (6). The envelope (E) protein is the primary surface-exposed protein on mature particles and is the principal target of the protective humoral immune system (7). The E protein is comprised of three domains (domain I [DI], DII, and DIII). DII contains several immunodominant epitopes, including the fusion loop (FL), which is a hydrophobic peptide that mediates the fusion of viral and host membranes in the late endosome. Domain III on E contains the putative cellular attachment domain (8). While several attachment factors have been postulated, specific entry receptors for YFV have not yet been identified. The virus enters host cells by receptor-mediated endocytosis, as the low pH of late endosomes triggers conformational changes in the E protein. These changes expose the FL, which inserts into the endosomal membrane, allowing the penetration of the RNA genome into the host cytoplasm for translation and replication. Although E protein is the primary target of neutralizing antibodies (9–12), nonstructural 1 (NS1) proteins can also elicit protective antibodies (13–15). Conversely, prM antibodies may confer the risk of antibody-dependent enhancement of infection by otherwise poorly infectious immature virions (16–18).

The YFV vaccine, based on a strain known as 17D, was created by serial passage and attenuation in the 1930s by Max Theiler (19) and is considered one of the most successful vaccines ever created. However, the production of 17D has changed little since its inception, resulting in a system of manufacturing and distribution that has been unable to keep pace with demand: ∼400 million people in areas of endemicity still require vaccination to achieve the herd-immunity threshold required to prevent the urban spread of YFV (20). Fractional dosing has been explored in outbreak settings when the vaccine supply is insufficient, but its consequences for the generation of durable, long-lasting protection are unknown (21, 22). YFV vaccine shortages stem principally from the limitations inherent in the legacy methods of vaccine strain propagation still being used. When outbreaks do occur in the setting of vaccine insufficiency, specific licensed antiviral treatments targeting YFV are not available.

Recently, potent neutralizing monoclonal antibodies (mAbs) against many viral targets have shown efficacy as potential treatments for highly pathogenic agents, including other flaviviruses. Several such antibodies targeting YFV have been described. A mAb designated A5 was identified using phage display technology and showed efficacy in an immunodeficient YFV-17D challenge model (23). A humanized mAb designated 2C9 showed benefit in hamsters against the Jimenez strain (24) and in AG129 mice against YFV 17D-204 challenge (25), supporting the proof of principle for antibodies as a medical countermeasure for YFV. Fully human mAbs with native heavy and light chain pairing, however, are preferred for use in human therapy. A human antibody designated TY014 has been tested in a phase 1 trial (26), and recently, other groups have reported the isolation of human anti-YFV mAbs (27, 28). Here, we isolated a panel of fully human mAbs targeting the E protein to identify candidate therapeutic antibodies. Competition-binding studies mapped these antibodies to several antigenic sites, one of which elicits antibodies that neutralize YFV. *In vitro* studies of the most potent neutralizing mAb, designated YFV-136, revealed that this antibody exerts its neutralizing activity at least partially at a postattachment step via binding to DII on the YFV E protein. Hydrogen-deuterium exchange mass spectrometry (HDX-MS) and neutralization escape virus selection established a key binding and functional epitope for YFV-136 in DII of the E protein. Passive transfer of YFV-136 mAb protected against lethal YFV challenge in a therapeutic setting in two small-animal models, Syrian golden hamsters and immunocompromised mice engrafted with human hepatocytes. These studies identify a potently neutralizing mAb targeting YFV and pave the way for the further development of this human mAb, YFV-136, as a possible candidate therapeutic agent.

## RESULTS

### Isolation of mAbs from YFV vaccine recipients.

**FIG 1:**
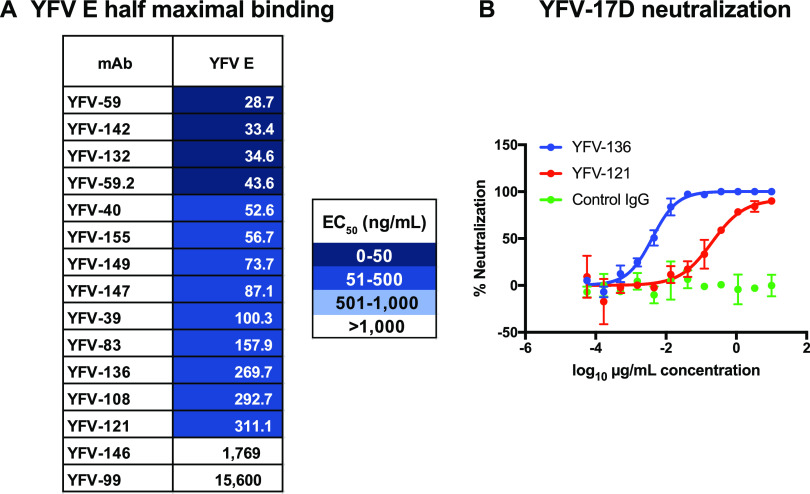
ELISA binding and FRNT neutralization by human mAbs targeting YFV E protein. (A) Half-maximal effective concentrations (EC_50_s) of antibody binding to YFV E as determined by ELISAs. Values were interpolated using a nonlinear regression model in Prism software. Data from a single experiment are shown, representative of results from three independent experiments. (B) Focus reduction neutralization test (FRNT) to assess the neutralization of YFV-17D by YFV-121 and YFV-136. Neutralization values were fit to a nonlinear regression model. Data from a single experiment are shown, representing results from at least two independent experiments.

Peripheral blood mononuclear cells (PBMCs) from four subjects who received a YFV vaccine previously (varying from months to years prior) were transformed *in vitro* with Epstein-Barr virus (EBV) to screen for YFV-reactive antibodies secreted by transformed memory B cells. We screened cell supernatants for binding to recombinant YFV E protein by an enzyme-linked immunosorbent assay (ELISA) and/or binding to YFV-17D-infected cells by flow cytometry. Cells secreting YFV-reactive antibodies were fused to a myeloma partner to generate hybridoma lines, which were cloned by flow cytometric cell sorting. Antibody was purified from serum-free hybridoma cell line supernatants by affinity chromatography. Using these methods, we isolated 15 mAbs from four YFV-immune subjects. These antibodies bound to recombinant E protein according to an ELISA with varying half-maximal effective concentrations (EC_50_s) for binding ranging from 29 to 15,600 ng/mL (Fig. 1A). Each of the antibodies isolated was tested for the ability to neutralize YFV-17D in a focus reduction neutralization test (FRNT) in Vero cells. While most antibodies did not neutralize YFV-17D infection, two mAbs showed inhibitory activity: YFV-121 was moderately neutralizing, with a half-maximal inhibitory concentration (IC_50_) of 202 ng/mL, and YFV-136 showed exceptional potency, with an IC_50_ below 10 ng/mL (Fig. 1B).

### Competition binding reveals antibodies that target several antigenic sites on the E protein.

**FIG 2:**
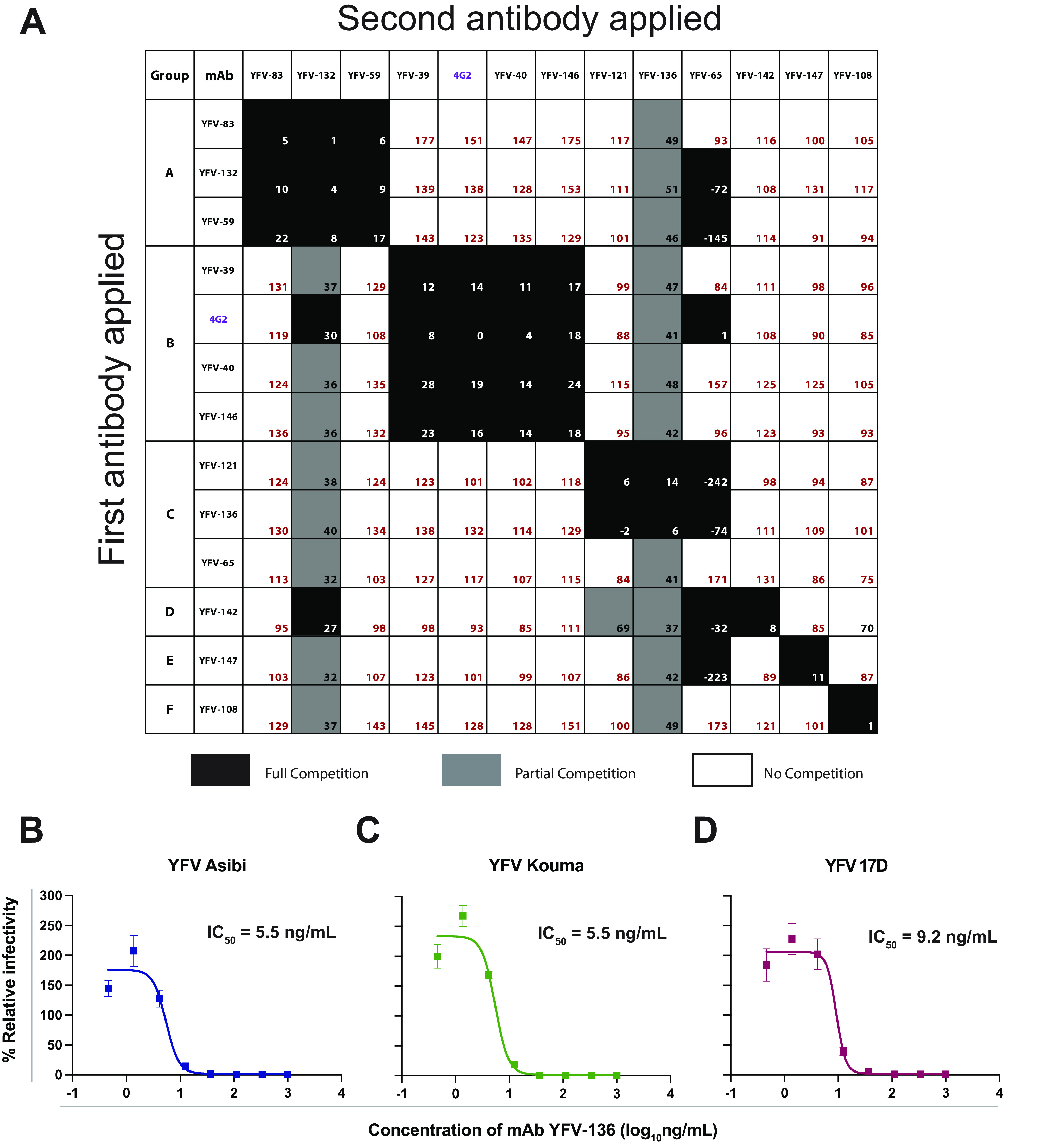
Competition binding and neutralization by human mAbs targeting YFV E protein. (A) Octet biolayer interferometry competition binding of human mAbs against YFV E. Antibodies listed from top to bottom were associated with immobilized YFV E protein, with antibodies shown from left to right tested for their ability to bind in the presence of the first antibody. Binding is expressed as a percentage of residual binding, with black boxes indicating complete competition, gray boxes indicating partial competition, and white boxes indicating no competition. Antibodies were clustered based on their competition profiles and labeled A to F. (B to D) Neutralization of diverse YFV strains assessed by a focus reduction neutralization test (FRNT). Values were fit to a nonlinear regression model using Prism software. Three independent experiments were performed in technical triplicate, with data from a single representative experiment shown.

We used biolayer interferometry (BLI) to perform competition-binding studies that enable the grouping of antibodies based on the major antigenic sites recognized (Fig. 2A). In this platform, antigen is loaded onto a biosensor tip, with two antibodies being sequentially flowed over the tip. If mAbs recognize nonoverlapping antigenic sites, both bind to the coated sensors when applied in sequence. If the binding of the first antibody applied to the antigen-coated sensor reduces or prevents the binding of the second antibody, the pair of mAbs likely binds to the same or an overlapping antigenic site. We included the previously described pan-flavivirus-reactive murine mAb 4G2 targeting the fusion loop (FL) for comparison (40). The human antibodies recognized six antigenic sites. One group of mAbs, including YFV-39, -40, and -146, competed for binding with 4G2, indicating that these mAbs target regions near the FL epitope on YFV E. The neutralizing mAbs YFV-121 and -136 grouped together, indicating that these mAbs target an overlapping antigenic site of neutralization vulnerability on YFV E. YFV-65 also competed for binding to E with YFV-121 and YFV-136, even though it did not neutralize YFV-17D when tested at concentrations as high as 10 μg/mL. These data suggest that there are multiple antigenic sites on YFV E, with at least one site being a target of potently neutralizing antibodies.

### Neutralization of wild-type YFV strains by mAbs targeting YFV E protein.

We next tested YFV-136 for its ability to neutralize wild-type (wt) YFV strains under biosafety level 3 (BSL-3) conditions. YFV-136 neutralized the Asibi and Kouma YFV strains as well as a different 17D vaccine strain with high potency (Fig. 2B to D). At lower antibody concentrations, we observed a modest enhancement of infectivity in this assay, possibly due to the aggregation of virions, as has been seen with other antiflavivirus antibodies in cells lacking Fcγ receptors (29).

### Identification of the antigenic site for mAb YFV-136 using HDX-MS studies.

**FIG 3:**
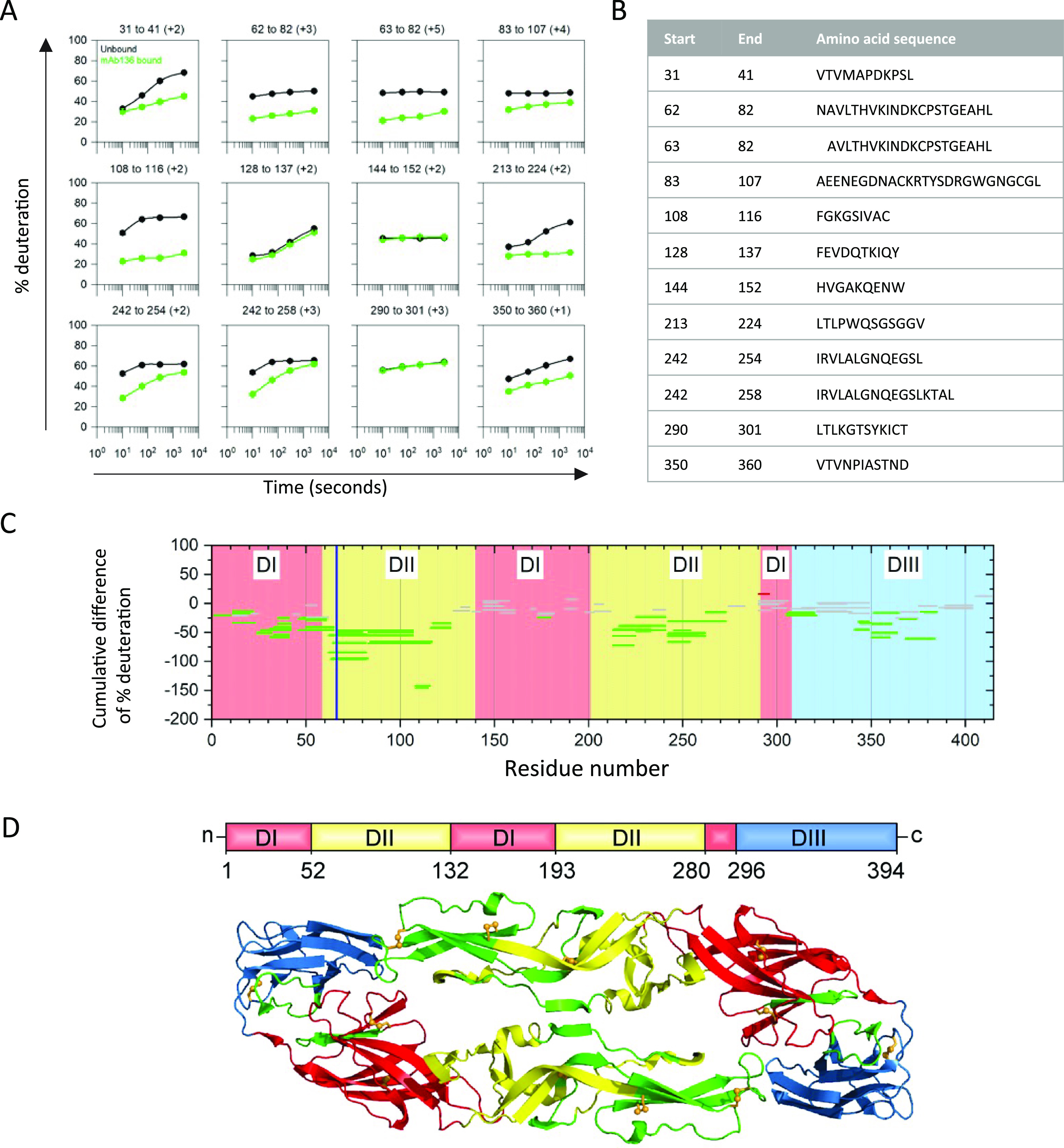
Epitope mapping for Fab YFV-136 by HDX-MS. (A) Representative kinetic plots for the 12 different peptides showing the effects of Fab binding by HDX. Black or green lines are for YFV E protein in the absence or presence of the mAb YFV-136, respectively. At the top of each panel are the residue numbers and charge states of the peptide. (B) Sequences and positions of each peptide. (C) Woods plots showing accumulated differences in percent deuteration (bound state − unbound state) across all time points for each analyzed peptide. The propagated error for the cumulative difference was calculated for each peptide, and 99% confidence intervals were calculated. Peptides whose differential exchange exceeds the 99% confidence interval are considered to show significant differences between the bound and unbound states and to be involved in binding. Peptides that do not show any significant differences between the bound and unbound states are in gray, whereas protected peptides are in green. The blue vertical line shows the location of the H67Y escape mutation identified in the studies shown in Fig. 4. (D) Protected peptides are shown in green on a ribbon representation of the YFV E dimer. The domains are indicated in red (DI), yellow (DII), and blue (DIII).

Using HDX-MS, a technique in which antibody binding reduces deuterium labeling of surface-exposed viral protein residues, we identified peptides on the YFV E protein that are occluded by the binding of YFV-136 Fab fragments (Fig. 3A). The start and end residues and the amino acid sequences of the representative E protein peptides that showed differential deuteration in the absence or presence of YFV-136 are shown in Fig. 3B. The results are summarized in a Woods plot in which the peptides showing a significant decrease in HDX are marked with green lines and the unaffected peptides are marked with gray solid lines (Fig. 3C). The HDX protection profile is mapped onto a cartoon representation of the YFV E dimer (Fig. 3D). E protein domain II (DII) near the fusion loop showed the most protection following YFV-136 Fab binding. Some protection against deuteration was also observed in the dimer interface and DIII.

### YFV-136 escape mutation studies identify a substitution at H67 that abrogates neutralization capacity.

**FIG 4:**
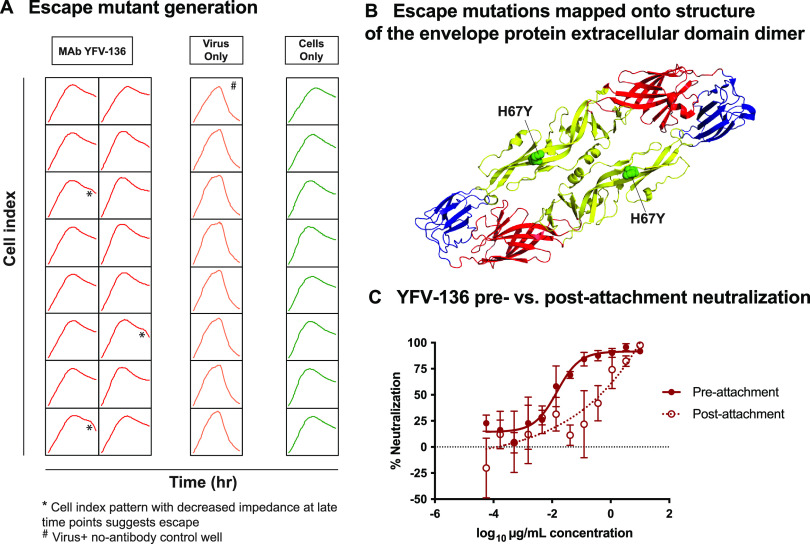
Critical residue for neutralization escape and mechanism-of-action studies for YFV-136. (A) Cell impedance measurements during the first round of YFV-17D escape mutant virus selection. Each box represents the cell impedance within a single well of a 96-well plate as a function of time. * indicates wells that exhibit a drop in cell impedance at late time points, suggesting viral escape in the presence of 5 μg/mL YFV-136. A single well marked with # was used as a control for cell culture adaptation. * and # wells were propagated once more in culture on the device and finally in 6-well culture dishes in the presence of 10 μg/mL YFV-136 (or no antibody for #) to allow viral outgrowth. Viral RNA was isolated, and the prM and E genes were amplified by RT-PCR using primers flanking the prM and E genes. These same primers, and two other primers targeting internal prM and/or E sequences, were used to sequence virus isolated from * and # wells by Sanger sequencing. These sequences were aligned in Geneious software to identify point mutations. (B) Escape mutant identified in panel A mapped onto the crystal structure of YFV E (PDB accession number 6IW5). Colors denote domain I (red), domain II (yellow), and domain III (blue). (C) Focus reduction neutralization test of YFV-136 before or after attachment of virus to host cells. Neutralization values were assessed using a nonlinear regression model in Prism software. Two independent experiments were performed in technical triplicate, with data from a single representative experiment shown.

To gain more insight into the epitope of YFV-136 in DII, we selected neutralization escape variants to identify functionally important interaction residues. To identify mutations in the YFV envelope protein that allow escape from YFV-136 neutralization, we used real-time cell analysis (RTCA). This high-throughput system monitors cell impedance and detects cytopathic effect (CPE) over time, allowing the identification of escape viruses by the observation of decreased cell impedance/CPE at late time points after incubating the virus with a neutralizing concentration of antibody. For these studies, YFV-17D was incubated with 5 μg/mL of YFV-136 in 16 wells of a 96-well plate. In 13 of 16 wells, complete neutralization and maintenance of cell monolayer integrity were observed throughout the study. However, 3 of 16 wells showed a late-CPE phenotype, suggesting the selection of variant viruses that subvert YFV-136 neutralization (Fig. 4A). Supernatants from these wells were harvested and again incubated with 5 μg/mL of YFV-136 on the RTCA instrument to confirm escape. In this second round, CPE developed rapidly, similar to a virus-only control, confirming the selection of a population of virus that is refractory to YFV-136 neutralization (data not shown).

The confirmation of viral escape using RTCA was followed by the outgrowth of virus in the presence of 10 μg/mL YFV-136. Viral RNA was isolated, and the prM and E genes were amplified and sequenced. In all three escape viruses, a single histidine-to-tyrosine substitution at position 67 on DII of YFV E was identified (Fig. 4B). This residue in the *b-*strand is conserved across all YFV genotypes, suggesting that this escape phenotype would likely be recapitulated in wild-type YFV strains. Overall, escape mutation studies identified a key residue in DII responsible for escape from YFV-136, suggesting that this mAb functions by binding an epitope including H67 on DII, consistent with the dominant region of protection from deuteration in the HDX studies.

### mAb YFV-136 neutralizes YFV-17D at a postattachment step.

Neutralizing antibodies can target different steps in the viral replication cycle, including, but not limited to, attachment, entry, or egress. To determine the mechanism of action for the most potently neutralizing antibody, YFV-136, we performed pre- and postattachment neutralization assays (Fig. 4C). In the preattachment inhibition assay, virus and antibody were premixed prior to addition to Vero cell culture monolayers. In the postattachment inhibition assay, virus was first incubated at 4°C with cells to allow attachment; afterward, the excess, unbound virus was washed away; and subsequently, antibody was added. YFV-136 neutralized infection in both assays, suggesting that at least part of its inhibitory activity occurs after viral attachment albeit with some diminished potency.

### mAb YFV-136 protects hamsters from lethal YFV challenge.

**FIG 5:**
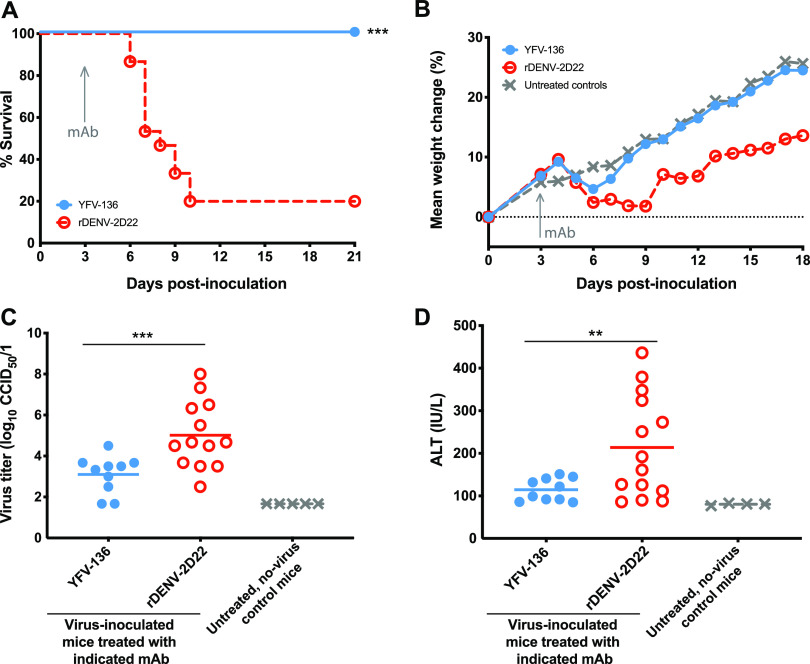
Syrian golden hamster challenge studies to assess YFV-136 therapeutic efficacy. (A) Kaplan-Meier survival curves of animals (YFV-136, *n* = 10; DENV-2D22 control, *n* = 15; uninfected controls, *n* = 5) treated with 50 mg/kg of YFV-136 or 20 mg/kg of the isotype control 3 days after inoculation with 200 50% cell culture infectious doses (CCID_50_) of the hamster-adapted YFV Jimenez strain. Statistical analysis was performed using a Wilcoxon log rank test. (B) Weights of YFV-infected animals treated with YFV-136 or isotype control mAb or uninoculated animals throughout the course of the study. (C) Serum virus titers (CCID_50_) were assessed 6 days after virus inoculation. One-way analysis of variance (ANOVA) with Dunnett’s multiple-comparison posttest was used to assess statistical significance. (D) Serum alanine aminotransferase (ALT) levels 6 days after inoculation were assessed as a proxy for liver damage. One-way ANOVA with Dunnett’s multiple-comparison posttest was used to assess statistical significance; *** indicates *p* < 0.001, ** indicates. *p* < 0.01.

Because YFV-136 is the most potently neutralizing antibody in our panel, we studied its activity *in vivo*. We first assessed the activity of YFV-136 in a model of YFV disease in Syrian golden hamsters. This model recapitulates many aspects of human YFV infections, including viscerotropism and liver infection, and has been used to assess the therapeutic efficacy of small molecules and antibody drugs (24, 30, 31). Prior to the *in vivo* study, we tested whether YFV-136 neutralized the hamster-adapted YFV Jimenez strain. mAb YFV-136 exhibited a 50% PRNT (PRNT_50_) value of 0.5 μg/mL when tested in a PRNT assay in Vero 76 cell monolayers using the hamster-adapted YFV Jimenez strain. Next, animals were administered 6 LD_50_s (half-maximal lethal doses) of the hamster-adapted Jimenez strain of YFV by an intraperitoneal (i.p.) route. At 3 days postinfection (dpi), 10 animals were treated with 50 mg/kg of body weight of YFV-136, and 15 animals were treated with 10 mg/kg of control dengue virus antibody 2D22 (DENV-2D22) (Table 1). Whereas 12 of 15 animals in the control group succumbed to infection, all animals in the YFV-136 group survived the 21-day study (Fig. 5A). Animals treated with YFV-136 showed transient weight loss after antibody treatment but quickly recovered and gained weight throughout the remaining course of the study (Fig. 5B). Viremia was assessed in all animals at day 6 after inoculation. While control mAb (DENV-2D22)-treated animals showed substantial viremia at day 6, animals treated with YFV-136 showed a significant reduction (Fig. 5C). Finally, we assessed the ability of YFV-136 to prevent YFV-induced liver damage in hamsters by measuring serum alanine aminotransferase (ALT). Whereas animals treated with an isotype control showed markedly increased serum ALT, animals treated with YFV-136 had lower ALT levels (Fig. 5D), suggesting that YFV-136 protects hamsters from hepatic damage induced by the hamster-adapted YFV strain Jimenez.

**TABLE 1:** Effect of delayed treatment (3 dpi) with mAb YFV-136 in a Syrian golden hamster model of YFV Jimenez strain infection and disease^*c*^

mAb treatment	Dose of mAb given at 3 dpi (mg/kg)	Virus given i.p. at 0 dpi	No. of alive/total no. of animals at 21 dpi	Mean day of death ± SD	Mean body wt change^*b*^ (g) ± SD	Mean serum virus titer (CCID_50_/mL) at 4 dpi ± SD	Mean serum ALT level (IU/L) at 6 dpi ± SD
YFV-136	50	YFV^*a*^	10/10	>21 ± 0.0**	−5.2 ± 6.7	4.1 ± 2.2	114 ± 26*
Isotype control (DENV-2D22)	10	YFV^*a*^	3/15	7.5 ± 1.4	−8.9 ± 9.9	5.4 ± 2.3	214 ± 120
Normal control	—	Sham	5/5	>21.0 ± 0.0**	3.0 ± 2.1**	1.7 ± 0.0**	80 ± 3*

a Hamster-adapted YFV Jimenez strain (24).

b Difference between weights at 4 and 5 dpi, representing the maximal weight change in this study.

c **, *P* < 0.001; *, *P* < 0.01 (compared to the control treatment).

### YFV-136 protects humanized mice from lethal YFV challenge.

**FIG 6:**
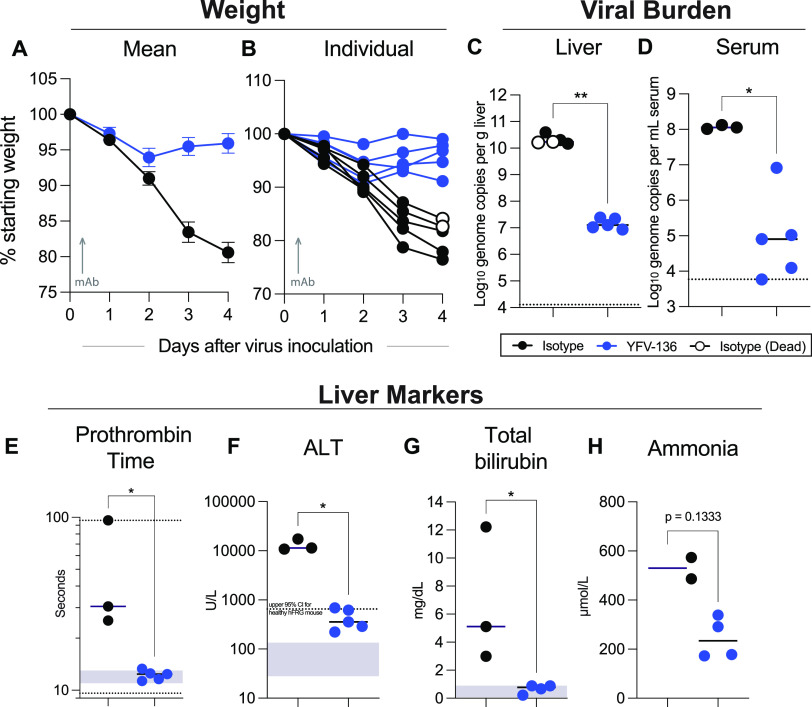
hFRG challenge studies to assess YFV-136 therapeutic efficacy. FRG mice engrafted with human hepatocytes (hFRG) were inoculated with 2 × 10^5^ focus-forming units (FFU) of wt YFV-DakH1279. Eight hours later, mice were administered a single 10-mg/kg dose of YFV-136 (*n* = 5) (blue points) or the isotype control (*n* = 5) (black points). Note that two of the control mAb-treated YFV-infected mice succumbed to infection at 4 dpi (denoted by open circles), and thus, some serum-based measurements were not available. (A and B) Weight loss showing mean values (A) and individual animal profiles (B). (C and D) Viral burdens at 4 dpi in the liver (C) and serum (D) as measured by RT-quantitative PCR (qPCR). (E to H) Serum biomarkers of hepatic injury at 4 dpi. Samples were tested for prothrombin time (E) and alanine aminotransferase (ALT) (F), total bilirubin (G), and ammonia (H) levels. For panels C to H, a Mann-Whitney test was performed (*, *P* < 0.05; **, *P* < 0.01). Bars denote median values. Dashed lines in panels C and D indicate the (lower and/or upper) limit of detection of the assay. Gray boxes indicate the reference range for each parameter in mice; for ALT, a dashed line is shown to denote the upper limit of “normal” for healthy hFRG mice at baseline. CI, confidence interval.

We recently developed a YFV infection model in mice engrafted with human hepatocytes (hFRG mice) (32). Immunodeficient hFRG mice have three genetic lesions (*Fah*^−/−^ [fumarylacetoacetate hydrolase knockout], *Rag2*^−/−^, and *Il2r*γ^−/−^ on a C57BL/6J background), which, together with specifically timed dietary modifications, facilitate the durable replacement of murine hepatocytes with transplanted human hepatocytes (33). YFV-infected hFRG mice developed disease that recapitulates many features of YF in humans, including massive hepatic infection and injury (32). Here, we tested the therapeutic activity of YFV-136 in this highly susceptible hepatotropic model. hFRG mice were administered a single 10-mg/kg dose of YFV-136 or isotype control mAb (DENV-2D22) 8 h after inoculation with 2 × 10^5^ focus-forming units (FFU) of wt YFV-Dakar (DakH1279), a highly pathogenic West African strain. By 4 dpi, all isotype mAb-treated hFRG mice displayed substantial signs of disease: two were dead, the other three were moribund, and all had lost 15 to 25% of their initial body weight (Fig. 6A and B). In contrast, hFRG mice that were inoculated with YFV and treated with YFV-136 mAb appeared healthy and exhibited minimal weight loss. The reduced disease observed in the YFV-136-treated group corresponded to significant (∼1,000-fold) reductions in YFV burdens in the serum and liver (Fig. 6C and D) and normal levels of liver synthetic function (as measured by the prothrombin time [PT]), hepatocyte damage (ALT), biliary function (bilirubin), and detoxification capacity (ammonium) (Fig. 6E to H). Thus, YFV-136 therapy given at 8 h postinfection was highly protective in the susceptible hFRG mouse model of YFV infection and liver disease.

## DISCUSSION

Yellow fever virus is a reemerging arbovirus with epidemic potential. While a highly effective live-attenuated vaccine is available for human use, safety and manufacturing concerns warrant new countermeasure development. Here, we isolated a panel of naturally occurring fully human mAbs that bind to the primary target of anti-YFV functional humoral immunity, the E glycoprotein. Two mAbs, YFV-121 and YFV-136, showed neutralization activity against YFV-17D, with YFV-136 showing exceptional potency with an IC_50_ of <10 ng/mL. The potency of YFV-136 represents one of the most potent mAbs against YFV ever isolated (23, 26), prompting us to study this mAb in detail. This mAb also neutralizes several wild-type strains of YFV. Both neutralizing mAbs YFV-121 and YFV-136 bind to overlapping antigenic sites as determined by competition binding, suggesting the recognition of a shared site of neutralization vulnerability. Antibody escape mutant virus studies identified H67 on DII as a critical residue for the function of YFV-136 within an epitope region in DII identified by HDX-MS studies. We show that YFV-136 functions to inhibit infection at least in part at a postattachment step in the viral life cycle. Finally, this mAb was highly efficacious in two different small-animal models of YFV infection even when administered after infection, suggesting that YFV-136 warrants further development as a therapeutic mAb for use in humans.

Previous studies support the concept that the administration of mAbs may be effective in reducing viral load and disease. The murine DII-specific mAb 2C9 and the murine-human chimeric mAb 2C9-cIgG exhibited therapeutic activity when administered 1 day after infection in interferon alpha/beta/gamma receptor-deficient strain AG129 mice challenged with the YF17D-204 vaccine (25) and against virulent YFV infection in an immunocompetent hamster model (24). A second murine-human chimerized mAb, designated 864-cIgG, recognizes DIII and neutralizes the YFV 17D-204 vaccine substrain but did not protect AG129 mice against 17D-204 infection, probably due to its low potency (reported as 10 μg/mL in a 90% plaque reduction neutralization test) (34). Other investigators have isolated fully human mAbs with similar ultrapotent (IC_50_ of <10 ng/mL) neutralizing activities (28), although animal protection studies have not been reported for these mAbs. One human antibody has been tested in a phase 1 clinical trial in which the mAb designated TY014 prevented viremia in 5/5 recipients, whereas only 1/5 placebo recipients lacked viremia 48 or 72 h following the administration of a live yellow fever vaccine (strain YF17D-204 [Stamaril]) (26); neutralizing potency and protection in animal studies for this mAb were not reported.

The antigenic site recognized by YFV-136, which lies proximal to the fusion loop (FL) on E protein domain II, has been previously implicated as being important for humoral immunity induced by YFV-17D vaccination (28). This recognition pattern is not restricted to human immune responses, as Ryman et al. also showed that some mAbs isolated from mice bind to a site proximal to H67, suggesting the broad immunodominance of this site (9). mAbs characterized previously by Wec et al. display a propensity for pairings of heavy and light chain genes encoded by the antibody variable genes *IGHV4-4* and *IGLV1-51*, suggesting that a public clonotype is elicited by YFV-17D vaccination (28). Our data complement these findings, as YFV-136 also uses this pairing. The neutralizing activity of YFV-136 is comparable to that of the most potent YFV antibodies reported (28). It is possible that the efficacy of YFV-17D hinges on its ability to elicit antibodies to this site since both neutralizing mAbs that we isolated from these donors are members of this public clonotype. However, the number of mAbs isolated here is not sufficient to make definitive conclusions in this regard. To date, few studies probing the humoral immune response to YFV have studied the circulating B cells of survivors of natural infection.

The epitope-mapping studies using HDX-MS and neutralization escape studies used here suggest most likely that the critical contacts of YFV-136 are focused on H67 and the region surrounding it on DII. This antigenic site is known in flaviviruses to be a site of vulnerability for potently neutralizing antibodies. For instance, we have observed a similar pattern of binding for the DII-reactive human mAb ZIKV-117 that potently neutralizes the related flavivirus Zika virus (35); ZIKV-117 also binds detectably to monomeric E protein, but it recognizes a quaternary epitope involving two protomers in the virus particle. Higher-resolution structural studies are needed to understand the interaction of YFV-136 with virus particles better and to identify a more complete binding footprint for YFV-136.

It is important to note that the antibodies highlighted here bind to monomeric YFV E protein. While we attempted to identify antibodies targeting quaternary structural epitopes present only on virions using a flow cytometric approach detecting antibodies binding to E protein expressed in YFV-infected cells, these efforts did not identify neutralizing mAbs, and the approach was not explored further. It is likely that functional mAbs targeting sites spanning one or more E protein dimers exist against YFV, as has been observed for many flaviviruses. In future work, a multipronged approach that employs screens for binding to protein and whole virions, as well as front-end neutralization screens, might help to reveal if immune humans possess some of this rare class of antibodies that exclusively recognize quaternary epitopes on virus particles.

## MATERIALS AND METHODS

### Generation of human mAbs.

Blood samples were obtained after written informed consent from four human subjects aged 20 to 47 years who were previously vaccinated with a YFV vaccine prior to travel. The studies were reviewed and approved by the Institutional Review Board of Vanderbilt University Medical Center. Peripheral blood mononuclear cells (PBMCs) were isolated from whole blood and transformed using Epstein-Barr virus (EBV), as previously described (36). Briefly, transformed B cells were expanded and cocultured with irradiated human PBMCs in 96-well plates. Cell supernatants were screened by an ELISA using recombinant YFV E protein (Meridian Life Sciences). Wells with positive reactivity were fused to a human-mouse myeloma cell line (HMMA 2.5) and plated by limiting dilution in 384-well plates. The resulting hybridomas were cloned by fluorescence-activated cell sorting (FACS) to produce clonal hybridoma cell lines. These clonal hybridoma cells were cultured in T-225 flasks containing serum-free medium, and mAb was purified from spent medium by affinity chromatography on an Äkta pure fast protein liquid chromatography (FPLC) instrument (Cytiva).

### Recombinant antibody expression and purification.

For animal studies, large-scale recombinant antibody production of YFV-136 was performed. RNA was isolated from the YFV-136 hybridoma line, and heavy and light chain genes were amplified using 5′ RACE (rapid amplification of cDNA ends) and sequenced using a Pacific Biosciences Sequel instrument. Variable regions of YFV-136 were cloned into a monocistronic full-length, human IgG1 expression vector (Twist Biosciences) for recombinant production. This expression vector was then transfected transiently into ExpiCHO cells for 7 to 8 days. Cell supernatants were harvested and filtered through 0.45-μm filters prior to purification. Purification was performed on an Äkta pure FPLC instrument (Cytiva) using HiTrap MabSelect SuRe columns (Cytiva) as described above for hybridoma-derived mAbs.

### ELISA binding of mAbs to YFV E protein.

Three-hundred-eighty-four-well plates were coated with 2 μg/mL of YFV E protein (Meridian Life Science) at 25 μL/well and incubated overnight at 4°C. Plates then were washed and blocked using Dulbecco’s phosphate-buffered saline (PBS) with Tween 20 (DPBS-T) containing 2% milk and 1% goat serum for 1 h at room temperature. Following a wash step, serial dilutions of antibody in DPBS were added to plates and incubated for 1 h at room temperature. To detect bound antibodies, alkaline phosphatase (AP)-conjugated goat anti-human IgG diluted 1:4,000 in DPBS-T containing 1% milk and 1% goat serum was added to plates for 1 h at room temperature and developed using AP substrate tablets diluted in 1 M Tris–0.3 mM magnesium chloride. Plates were developed in the dark for 1 h and read on a BioTek plate reader at 405 nm. Binding curves were interpolated in Prism software (GraphPad) using nonlinear regression analysis.

### YFV-17D focus reduction neutralization test.

A focus reduction neutralization test (FRNT) was performed as previously described, with minor amendments. Briefly, 96-well plates were seeded with Vero cells at 2.5 × 10^4^ cells/well and incubated overnight (41). The following day, serial dilutions of antibody were mixed with 10^2^ FFU YFV-17D and incubated at 37°C for 1 h. A total of 30 μL/well of the virus-antibody mixture was then added to Vero cell culture monolayers, and the mixture was incubated at 37°C for 1 h. Without washing, 110 μL per well of an overlay containing a 1:1 mixture of 2.4% methylcellulose and 2× Dulbecco’s modified Eagle medium (DMEM) with 4% fetal bovine serum (FBS) was added to plates, which were then incubated for 72 h at 37°C in 5% CO_2_. To stain foci of virus infection, cells were fixed with 1% paraformaldehyde for 1 h at room temperature, washed, and permeabilized using permeabilization buffer (0.1% saponin and 0.1% bovine serum albumin [BSA] in DPBS) for 10 min. Cells were then stained with 1 μg/mL of pan-flavivirus murine mAb 4G2 in permeabilization buffer for 1 h at room temperature. After two washes, goat anti-mouse IgG-horseradish peroxidase (Southern Biotech) diluted 1:1,000 in permeabilization buffer was added to cells, and the mixture was incubated for 1 h at room temperature. Foci were developed using TrueBlue peroxidase and counted using a spot counter instrument (ImmunoSpot; CTL). Focus counts were normalized to that of a virus-only control, and neutralization curves were interpolated in Prism software using nonlinear regression analysis.

### Wild-type and 17D YFV strain FRNT.

An FRNT was performed as described above, with the following exceptions. One hundred microliters containing 200 FFU of virus was mixed with 100 μL of serially diluted mAb and incubated at 37°C for 1 h. One hundred microliters of the virus-mAb mixture was then added to Vero cells in a 96-well plate format and incubated at 37°C for 1 h, followed by the addition of an overlay and incubation at 37°C for 2 days. Cells were then fixed with 4% paraformaldehyde (final concentration) for 30 min, permeabilized, stained, and analyzed as described above.

### Pre- and postattachment neutralization of YFV-17D.

Pre- and postattachment neutralization assays were performed as previously described (37). For preattachment studies, 600 FFU YFV-17D was mixed with serial dilutions of antibody for 1 h at 37°C. Cells and virus-mAb mixtures were then prechilled for 15 min prior to the addition of mixtures to cell monolayers for 1 h at 4°C. Cells were then washed three times and incubated with prewarmed DMEM for 15 min prior to the addition of a methylcellulose overlay containing DMEM. For postattachment studies, cell monolayers were first incubated with virus for 1 h at 4°C. Cells were then washed and incubated with serial dilutions of antibody for 1 h at 4°C. Excess antibody was then washed off, and cells were incubated for 15 min at 37°C with DMEM prior to the addition of the overlay. Foci were enumerated as described above for the focus reduction neutralization test.

### Biolayer interferometry competition-binding assay.

Competition-binding studies were performed using a biolayer interferometry instrument (FortéBio Octet HTX). HIS1K sensortips were preincubated in kinetic buffer (Pall) for 10 min. After a 60-s baseline step, His-tagged YFV E protein (Meridian Life Science) was associated with the tips at 5 μg/mL for 60 s. Readings were again set to the baseline for 60 s, followed by the association of the first antibody at 25 μg/mL for 600 s to achieve complete saturation. Tip readings were again set to the baseline, and the tips were then dipped into wells containing a second antibody at 25 μg/mL for 180 s. Data were analyzed using FortéBio data analysis software. Data from all steps were normalized to a buffer-only control, and antibodies were grouped using Pearson correlation statistical analysis.

### Hydrogen-deuterium exchange mass spectrometry. (i) Soluble recombinant E protein used for HDX studies.

A synthetic DNA fragment encoding residues 123 to 680 (TLV…EGSS) of yellow fever virus strain 17DD-Brazil (GenBank accession number AAZ07885.1) E protein was inserted downstream of a modified human interleukin-2 (IL-2) signal peptide (MARMQLLSCIALSLALVTNSV). The construct was also modified at the C terminus of the envelope region to contain a small linker, a tobacco etch virus (TEV) protease site, and a 6-His tag (GSTGGSENLYFQGHHHHHH). The fusion construct was inserted into an AgeI-NotI-cut pFM1.2R vector (38) by Gibson assembly to lie downstream of the cytomegalovirus (CMV) promoter. Recombinant E protein was produced by transient transfection of Expi293F cells using an ExpiFectamine 293 transfection kit (Thermo Fisher Scientific). Cell supernatants were harvested 4 days after transfection and then concentrated before exchange into 2× PBS at pH 6.5 and finally into 2× PBS at pH 8.0. The soluble recombinant E protein was recovered by 6-His affinity chromatography on Ni-nitrilotriacetic acid (NTA) agarose (G-Biosciences) and purified by size exclusion chromatography on a Superdex S200 Increase column (Cytiva).

### (ii) Peptide mapping.

To prepare for the acquisition and analysis of hydrogen-deuterium exchange (HDX) data, the YFV E protein was digested with two acid proteases (immobilized pepsin followed by immobilized acid protease from fungal type XIII) to achieve better sequence coverage. To effectively reduce the protein and denature it, a quench solution containing Tris(2-carboxyethyl)phosphine hydrochloride (TCEP) and guanidine hydrochloride (GdnHCl) was added. The quenching conditions were 1:1 dilution of the HDX reaction mixture volume (100 μL) with quench buffer containing 500 mM TCEP and 4 M GdnHCl (pH 2.4) (resulting in 250 mM TCEP and 2 M GdnHCl at pH 2.6), with a 3-min incubation at 25°C. A peptide map (in triplicate) of the digest was generated by liquid chromatography-tandem mass spectrometry (LC-MS/MS) using a Maxis-II-HM mass spectrometer (Bruker Daltonics, Billerica, MA). YFV E protein (100 pmol) was injected into the LC-MS system where the protein was digested, and the resulting peptides were captured and desalted by a C_8_ trap column (2.1 by 20 mm, Zorbax Eclipse XDB-C_8_ trap; Agilent), followed by loading onto a C_18_ column (2.1- by 50-mm, 2.5-μm Xselect-CSH; Waters, Milford, MA) and elution into the mass spectrometer. The mass spectrometer was operated in a data-dependent fragmentation mode with monitoring of the high-abundance peptides. Data were analyzed by Byonic (Protein Metrics, Santa Carlos, CA, USA) for sequencing and accurate precursor mass (±5 ppm), and the peptides were also curated manually.

### (iii) HDX experiment.

YFV E was equilibrated without or with antibody (1:2 antibody) in PBS (pH 7.4) in H_2_O overnight at 4°C and reequilibrated at 25°C for 30 min before starting the HDX experiment. The exchange-in with D_2_O (PBS prepared in D_2_O) in the absence (10 μM YFV E) or presence (20 μM antibody [1:2 antigen-to-antibody ratio]) of antibody was initiated by diluting the protein solutions (10 μL) 10-fold with D_2_O in PBS at 25°C (90 μL; pH 7.4). HDX was measured at 0 s (undeuterated control), 10 s, 60 s, 300 s, and 2,700 s at 25°C. For the undeuterated control, the conditions were the same except that the added buffer solution was H_2_O instead of D_2_O. The HDX was quenched by adding an equal volume (100 μL) of quench buffer equilibrated at 25°C, followed by mixing and incubation at 25°C for 3 min. The quenched sample was digested by passing it through a custom-packed column (2 mm by 20 mm) of immobilized pepsin beads followed by a column of immobilized fungal XIII beads (2 mm by 20 mm) at a 200-μL/min flow rate. The resulting peptides thus generated were captured and desalted on a C_8_ column by washing with 0.1% formic acid in water for 4.7 min. Desalted peptides were loaded onto a C_18_ analytical column where peptides were separated using a gradient of acetonitrile (ACN) in 0.1% formic acid (most peptides eluted during the linear part of the gradient from 5 min [4% ACN] to 15 min [40% ACN]). To minimize back exchange, the trap and analytical columns were kept in an ice slush, while protease columns were kept at room temperature. The isotope distributions of the exchanged peptides were measured with a Maxis-II-HM mass spectrometer (MS-only mode) for duplicate samples.

### (iv) HDX data analysis.

LC-MS HDX data acquisition (retention time, isotopic distribution, and observed *m/z*) was directed by the peptide map, and data were analyzed by HDExaminer (v2.5.0, 64-bit; Sierra Analytics). The maximum deuterium level was set to 90%, and the data were displayed as kinetic plots for each peptide for HDX. Only those peptides that provided a good signal-to-noise ratio at all time points and for both states were included in the analysis. Ultimately, 107 unique peptides covering 95% of the sequence of the YFV E protein were analyzed. The average peptide length was 14 amino acids, and the average residue level redundancy was 4. To elucidate those regions where HDX changed with statistical significance upon antibody binding, the mean cumulative difference (bound − unbound) across all time points for each peptide was calculated and plotted as a Woods plot. To identify significant differences upon binding, the propagated error for the cumulative percent deuteration difference for each peptide was calculated using the standard error of the mean, and a 99% confidence interval was determined (2 degrees of freedom; two-tailed distribution). Peptides that showed no change are marked in gray, and peptides that showed significant differences between the bound and unbound states are highlighted based on protection (green) or exposure (red).

### Generation and analysis of YFV-17D escape mutations.

In a U-bottom 96-well plate, 25 μL mAb YFV-136 IgG protein at 5 μg/mL was premixed with 25 μL YFV-17D (39) diluted 1:10 (∼5,000 FFU) in DMEM without FBS and incubated for 1 h at 37°C. This procedure was done in 16 separate wells within the 96-well plate. Virus also was mixed with DMEM alone and passaged throughout the study to control for substitutions that arise from cell culture adaptation. Fifty microliters of the virus-antibody mixture and controls were added to Huh7.5 cell culture monolayers in 96-well ePlates (Agilent) and incubated for 1 h at 37°C. One hundred microliters of DMEM containing 5% FBS was then added to each well, plates were placed back onto an xCELLigence instrument (Agilent), and cell monolayers were monitored for delayed CPE. Cell supernatants in wells with a delayed-CPE phenotype, as well as a virus-only control, were subjected to a repeat of this assay to confirm viral escape. Once escape was confirmed, 6-well plates containing confluent Huh7.5 cell monolayers were inoculated with 100 μL/well of escape virus or a virus control in the presence of 10 μg/mL of YFV-136 for the outgrowth of escape virus. Virus was harvested from 6-well plates, and RNA was isolated using the Qiagen virus RNA isolation kit. E and prM genes from isolated RNA were reverse transcribed to cDNA and PCR amplified using primers flanking the prM and E genes (one-step reverse transcription-PCR [RT-PCR] kit). Genes were then sequenced by Genewiz using overlapping primers that give coverage across prM and E. The control virus sequence was aligned to the 17D reference genome sequence to confirm that mutations did not result from adaptation to cell culture.

### Syrian golden hamster challenge studies.

The Syrian golden hamster model used for these studies has been described previously (24). Thirty female Syrian golden hamsters (LVG/Lak strain) supplied by Charles River were used. Hamsters were randomized by weight to experimental groups and individually marked with ear tags. For challenge studies, hamsters were challenged at day 0 with 200 50% cell culture infectious doses (CCID_50_) of the hamster-adapted YFV Jimenez strain by bilateral intraperitoneal injections in a total of 0.2 mL. Three days after virus inoculation, hamsters were dosed with 50 mg/kg of recombinant YFV-136 (rYFV-136) (1-mL total volume) or 10 mg/kg of the rDENV-2D22 control and monitored for weight loss and clinical manifestations for 21 days. Blood samples were taken at days 4 and 6 to assess viremia and ALT. Any surviving animals were humanely euthanized at the experimental endpoint.

### Measurement of hamster serum aminotransferase.

ALT (serum glutamic pyruvic transaminase [SGPT]) reagent (Teco Diagnostics, Anaheim, CA) was used, and the protocol was altered for use in 96-well plates. Briefly, 50 μL of the aminotransferase substrate was placed into each well of a 96-well plate, and 15 μL of the sample was added at timed intervals. The samples were incubated at 37°C, after which 50 μL of color reagent was added to each sample, and the mixture was incubated for 10 min as described above. A volume of 200 μL of a color developer was next added to each well, and the mixture was incubated for 5 min. The plate was then read on a spectrophotometer, and aminotransferase concentrations were determined according to the manufacturer’s instructions.

### CCID_50_ assays to assess hamster viral burdens.

The virus titer was quantified using an infectious cell culture assay in which a volume of either the tissue homogenate or serum was added to the first tube of a series of dilution tubes. Serial dilutions were made and added to Vero cell monolayer cultures. Ten days later, CPE was used to identify the endpoint of infection. Four replicates were used to calculate the CCID_50_ of virus per milliliter of plasma or gram of tissue.

### Mouse studies.

All mouse experiments were conducted under a Washington University School of Medicine Institutional Animal Care and Use Committee-approved protocol in compliance with the Animal Welfare Act. Female hFRG mice were generated by Yecuris Corporation and maintained according to their specific care and use guidelines (see reference 32 for additional details). Only mice with human albumin levels of ≥5.0 mg/mL in plasma (indicative of ≥70% engraftment) were used for this study. hFRG mice were continued on their regular diet of PicoLab high-energy mouse diet 5LJ5 chow (LabDiet) and 3.25% (wt/vol) dextrose–water during infection experiments. Mice were inoculated via retro-orbital injection of 50 μL containing 2 × 10^5^ FFU of wt YFV-DakH1279, obtained from the World Reference Center for Emerging Viruses and Arboviruses, and passaged once in Vero-CCL81 cells. Antibodies were given as a single treatment of 10 mg/kg dose, diluted in PBS in a 100-μL total volume, and given by the i.p. route at 8 h postinfection. The DENV-2D22 mAb was used as a control. Euthanasia was performed via ketamine overdose and thoracotomy. Blood was collected via aspiration from the cardiac chambers, and PT was measured on a Coagucheck meter (Roche). Perfusion of the entire vascular tree was then performed with saline prior to liver collection. After centrifugation, serum was mixed 1:9 with 10% Triton X-100 in Hanks’ balanced salt solution (HBSS) (1% final volume of Triton X-100) and then incubated at room temperature for 1 h to inactivate infectious virus (32). ALT, bilirubin, and ammonia were analyzed using the Catalyst Dx chemistry analyzer (Idexx Laboratories). Some specimens required dilutions to achieve an ALT value within the analytical measurement range; in these instances, dilution was performed in HBSS, and the value was corrected by the final dilution factor. RNA extraction and viral load analyses were performed as described previously (32) using the KingFisher Flex instrument (Thermo Fisher) and the TaqMan RNA-to-CT 1-step kit (Thermo Fisher) on the QuantStudio 6 Flex real-time PCR system with the following primers: forward (F) primer 5′-AGGTGCATTGGTCTGCAAAT-3′, reverse (R) primer 5′-TCTCTGCTAATCGCTCAAIG-3′, and probe (P) 5′-/56-FAM/GTTGCTAGGCAATAAACACATTTGGA/3BHQ_1/-3′; FAM indicates fluorescein amidite dye; BHQ indicates Black Hole Quencher (BHQ™) dye.
